# The allometry of the arcuate body in the postembryonic development of the giant house spider *Eratigena atrica*

**DOI:** 10.1007/s10158-018-0208-4

**Published:** 2018-03-10

**Authors:** Teresa Napiórkowska, Jarosław Kobak

**Affiliations:** Department of Invertebrate Zoology, Faculty of Biology and Environmental Protection, Nicolaus Copernicus University, Lwowska 1, 87-100 Toruń, Poland

**Keywords:** Allometry, Arcuate body, Central nervous system, Postembryonic development, Spider

## Abstract

The brain of arachnids contains a special neuropil area called the arcuate body (AB), whose function has been widely discussed. Its growth and proportion in the brain volume during postembryogenesis have been investigated only in several spider species. Our allometric study is aimed at determining to what extent the development of the AB in *Eratigena atrica*, a spider with unique biology and behaviour, is similar to the development of this body in other species. We put forward a hypothesis of allometric growth of this body in relation to the volume of the central nervous system (CNS) and its neuropil as well as in relation to the volume of the brain and its neuropil. The analysis of paraffin embedded, *H* + *E* stained histological preparations confirmed our hypothesis. The AB developed more slowly than the CNS and the neuropil of both the brain and the CNS. In contrast, it exhibited positive allometry in relation to the volume of the brain. This body increased more than nine times within the postembryonic development. Its proportion in the brain volume varied; the lowest was recorded in larvae and nymphs I; then, it increased in nymphs VI and decreased to 2.93% in nymphs X. We conclude that in *Eratigena atrica*, the AB develops differently that in orb-weaver and wandering spiders. There is no universal model of the AB development, although in adult spiders, regardless of their behaviour, the proportion of this area in the brain volume is similar.

## Introduction

The arthropod brain contains neuropil areas having special functions. For example, in insects, they may be responsible for walking (Strauss 2002), sounds (Hoffmann et al. 2007), long-term olfactory memory (Wu et al. 2007) and polarized vision (Heinze and Homberg 2007). Such areas also occur in Chelicerata. One of them, located in the back of the protocerebrum, is known as the arcuate body (AB) (Strausfeld et al. 1993, 2006; Strausfeld 1998; Homberg 2008) and previously also as the central body (e.g. Babu 1965; Satija et al. 1970a, 1980; Babu and Barth 1984; Wegerhoff and Beridbach 1995). The later term is still in use (Foelix 1996; Barth 2002; Hwang and Moon 2003; Park and Moon 2013; Park et al. 2013), though, due to the lack of the homology of this structure with the “central complex” structures of insects (Homberg 1987; Hanesch et al. 1989; Strausfeld 1999) and malacostracans (Utting et al. 2000; Loesel et al. 2002), the term “arcuate body” seems to be more suitable (Strausfeld et al. 1993, 2006; Strausfeld 1998; Homberg 2008).

**Fig. 1:**
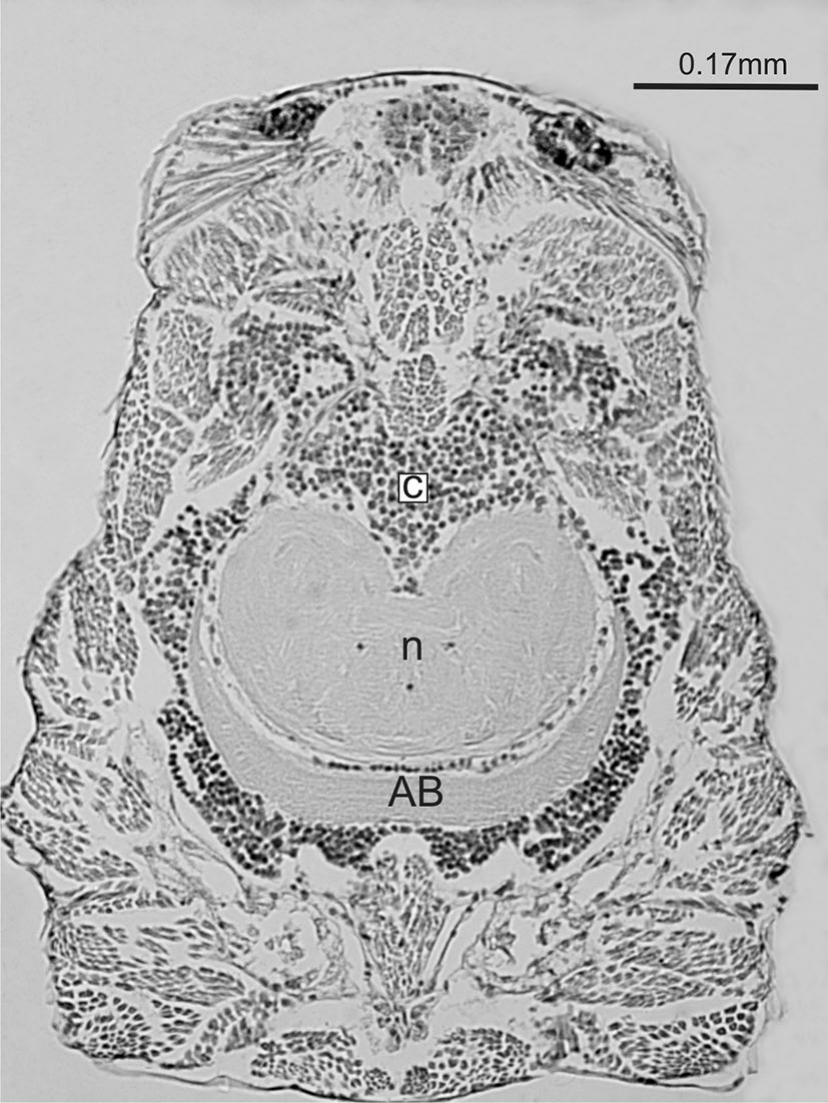
Horizontal section through the prosoma and brain of *Eratigena atrica* nymph II; *AB* arcuate body, *c* cortex, *n* neuropil

AB has been shown in many chelicerates: Pycnogonidae, Xiphosurida, Scorpionida, Solifugae, Thelyphonida, Opilionida and Araneida (Babu 1965; Loesel et al. 2002; Harzsch et al. 2005; Loesel et al. 2011). It is vestigial or absent only in Acari (Ioffe 1963). This unpaired, crescent-shaped neuropil mass is located in the back region of the brain. It is not built into the protocerebrum, but occupies the dorsal surface, lying transversely to the long body axis (Fig. 1). The crescent horns point forward (Satija et al. 1970a, b; Satija et al. 1980; Babu and Barth 1984; Wegerhoff and Beridbach 1995; Barth 2002; Kovoor et al. 2005; Loesel et al. 2011; Park et al. 2013). AB is composed of lobes. In spiders, there are two lobes of similar thickness: anterodorsal and posteroventral (Babu and Barth 1984; Barth 2002; Doeffinger et al. 2010; Park et al. 2013), although Babu (1965) distinguished three lobes in *Poecilotheria*: anterior, central and posterior (situated ventrally to the others). In *Lycosa tarentula*, these two lobes are not set in the same horizontal plane: 2/3 of the inner surface of the posteroventral lobe is covered by the anterodorsal one (Kovoor et al. 2005). Each lobe has a layered structure. Five layers were distinguished in *Lycosa tarentula* by Kovoor et al. (2005) in the anterior lobe, and only two in the posterior one. Their texture results from branched fibres oriented in different directions, and from the accumulation of numerous synaptic connections. The 50-μm-thick brain cortex covers the entire surface of the anterodorsal lobe and only 50% of the posteroventral one. The AB cortex contains several neuron types. Their projections form a multi-column, palisade-like organization of the AB.

The function of the arachnid AB has long been discussed. It has been suggested to play a role in the web building, as lesions in the cortex close to the AB led to abnormalities in the web structure of web-weaving spiders (Witt 1969) and the beginning of web production in *Argiope aurantia* coincided with the formation of AB (Babu 1975). However, the hypothesis that the AB is best developed in web-weaving spiders has not been confirmed. Weltzien and Barth (1991) have revised the view that the AB is the main centre of web spinning. They compared the volume of different brain regions, including AB, in four spiders with different behaviour. The proportion of the AB volume in the brain volume was always similar and relatively small, ranging from 3.1 to 5.1%. Thus, the AB does not only coordinate web weaving, especially as it has also been found in non-weaving arachnids, for example, scorpions (Babu 1965). Strausfeld et al. (1993) showed the association of the AB with the optical system of spiders and other arachnids. In spiders, the optical pathway leading from the principal antero-median eyes does not end in the first optical neuropil, running further to two other neuropil masses, one of them being the AB (Foelix 1996).

Numerous studies of the central nervous system (CNS) of spiders have focused on the AB presence and structure (e.g. Babu 1965, 1969, 1975; Babu and Barth 1984; Wegerhoff and Beridbach 1995; Park and Moon 2013), but have largely ignored its morphometry. This omission may have resulted from the fact that for many years, the AB function has not been fully understood. Weltzien and Barth (1991) performed a morphometric analysis of the brain and its centres in four juvenile and/or adult spiders from different families: *C. salei*, *N. clavipes*, *P. regius* and *Ephebopus* sp. Satija et al. (1969, 1970a, b) evaluated the volume of the AB in ecribellate (*Crossopriza lyoni* and *Cyrtophora citricola*) and cribellate spiders (*Stegodyphus pacificus*, *Oecobius putus*, *Filistata poonaensis*). In *C. lyoni*, *C. citricola* and *S. pacificus*, the AB proportion in the brain volume was 5–7%, and in *O. putus* and *F. poonaensis*, 35%. The latter result differs considerably from the results obtained by Weltzien and Barth (1991) and by Satija et al. (1969, 1970b) and is not fully credible. However, it cannot be excluded that in some spiders, the AB occupies a greater part of the brain than in the species examined so far.

To shed new light on the issue, we conducted a morphometric study of the AB in *Eratigena atrica* (Agelenidae). In Agelenidae, the AB volume and proportion in the brain volume have not been determined yet. We assumed that our results might be different from those obtained by Weltzien and Barth (1991) and Satija et al. (1969, 1970a, b) due to the unique behaviour of this species. *E. atrica* builds specific flat webs with a funnel at one end, where the spider lurks. The web structure is simple and does not require great precision, leg coordination or spatial orientation, i.e. features found in typical web-weaving spiders. Moreover, *E. atrica* often leaves their webs to hunt. Thus, it may be viewed as intermediate between web-weaving and wandering spiders. We put forward a hypothesis of an allometric growth of this body in relation to the volume of the entire central nervous system and its neuropil as well as to the volume of the brain and its neuropil. We assumed that, if the AB is responsible for a particular behaviour type, such as web weaving or locomotion, there will be an association between the life style of particular spider species and the relative size and/or allometric growth of their AB. *Eratigena atrica*, occupying an intermediate position between typical web-weaving and wandering species, should exhibit intermediate parameters of the AB growth. Also, the role of the AB in walking would result in the increase in the importance of this structure when developing individuals start to move actively. Therefore, we compared our results with those obtained for spiders differing in behaviour type by Babu (1975) and Weltzien and Barth (1991).

## Materials and methods

The study involved *Eratigena atrica* C. L. Koch (Agelenidae) from our laboratory culture, established at the end of August 2014. Sexually mature spiders (34 females, 18 males) were collected in the vicinity of Toruń and Chełmża (Poland) and transferred to a dark laboratory room with temperature of 21–23 °C and relative humidity of 70%. Each individual was placed in a 500-ml glass jar which contained water-soaked cotton ball and fed twice a week with larvae of *Tenebrio molitor*. Several weeks later, sexually mature males were introduced into the jars with females. The procedure was repeated twice, each time with a different male. Females laid first cocoons 3–4 weeks after the last copulation. The cocoons were cut open to remove eggs, subsequently incubated in conditions optimum for embryonic development of this spider species, i.e. at the temperature of 21–23 °C and relative humidity of 70% (Jacuński et al. 1994; Jacuński and Wiśniewski 1997). In the next step, we randomly selected 15 larvae (called postembryos) (Downes 1987; Wolff and Hilbrant 2011) from a large population and fixed them on the second day after hatching. The remaining larvae were further cultured. Subsequent stages were kept in separate jars and fed twice a week: juveniles with newly laid eggs of *E. atrica*, while later stages, with larvae of *Tribolium* sp. and *Tenebrio molitor*. The spiders were monitored every day to capture molts, which indicated their transition to the next developmental stage. All spiders were fixed on the second day after casting off the exuvium. In total, we fixed 165 individuals, 15 of each developmental stage (from larvae to nymphs X). At this point, we decided to close the culture because we already have one male among the 15 specimens. We assumed that the remaining 14 individuals were females, which needed one more molt to reach sexual maturity. Eventually, though, only females were subjected to the morphometric analysis.

Spiders were fixed with Bouin solution, composed of saturated picric acid, formalin and acetic acid (15, 5 and 1 ml, respectively). The fixation time was 72 h, but after 48 h, when the tissues were initially fixed, spiders had all appendages removed. Older stages also had their opisthosoma removed for better penetration of the tissues with the fixative.

The fixed tissues were washed in ethanol of increasing alcohol content (from 70 to 100%) for dehydration. The dehydrated material was passed through a mixture of absolute alcohol and xylene (1:1), then twice through pure xylene. Next, it was transferred to a mixture of xylene and paraffin (1:1) and finally to pure paraffin. The paraffin blocks were cut into 7 μm horizontal sections using HM 355 S Microm rotary microtome. The sections were placed on slides, dried, stained with Mayer hematoxylin and eosin and embedded in euparal.

**Fig. 2:**
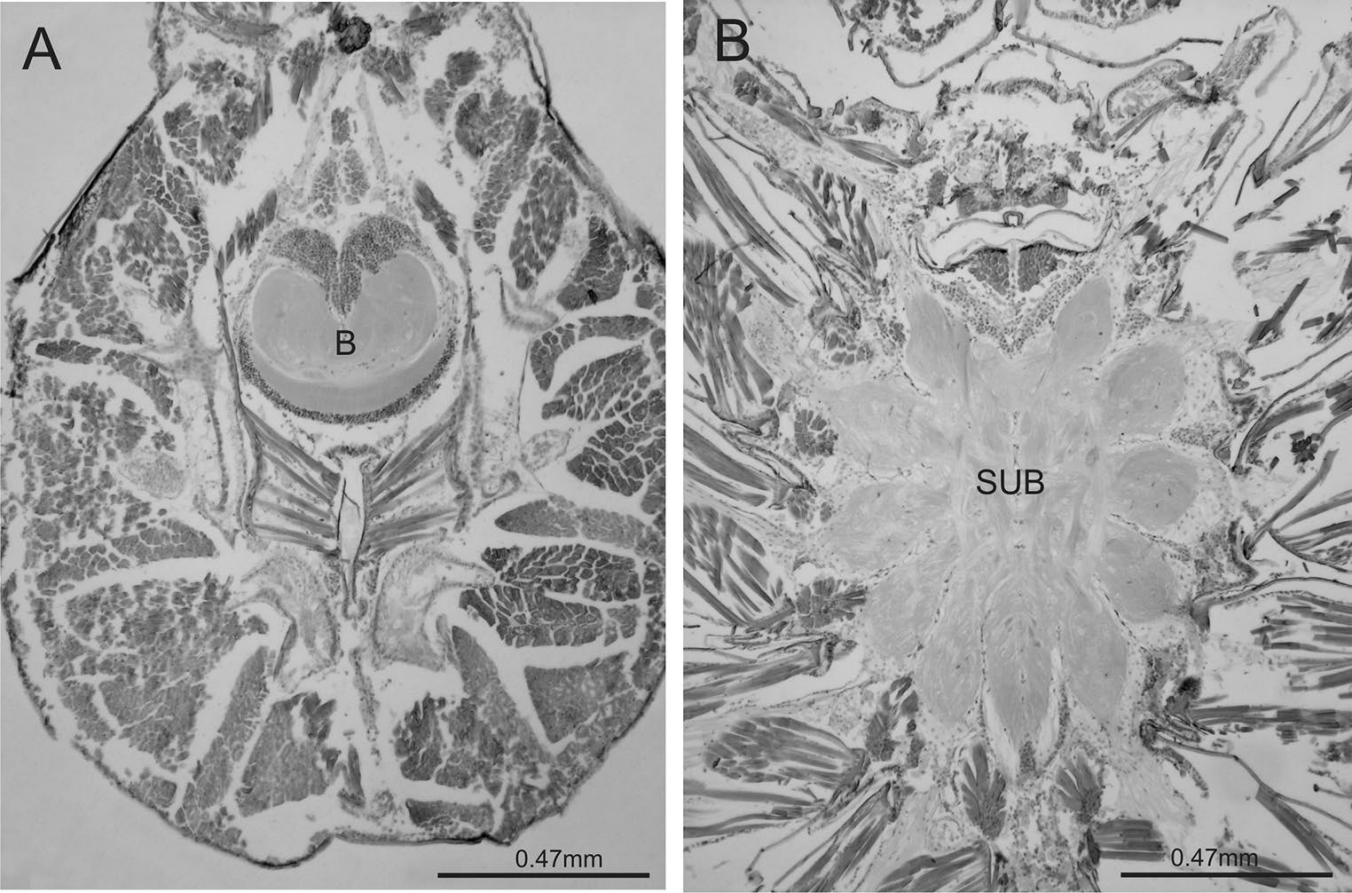
Horizontal sections through the prosoma, brain (**a**) and subesophageal ganglion (**b**) of *Eratigena atrica* nymph VI; *B* brain, *SUB* subesophageal ganglion

Morphometric analysis was performed on ten specimens of each developmental stage, for which we obtained a complete series of stained sections. Each section was photographed under a microscope with a digital camera connected to the computer. ImageJ software (freeware by W. S. Rasband, US National Institutes of Health, Bethesda, Maryland, USA, https://imagej.nih.gov/ij/) was used to determine the surface of the central nervous system (consisting of the brain and subesophageal ganglion) (hereafter referred to as the CNS) in each slide (Fig. 2a, b). If a series was incomplete (ca. 2% of sections were missing), we averaged the measurements of the previous and next sections. The procedure was similar for determining the surface of the brain (cortex and neuropil). The position of the oesophagus visible on the sections was considered a criterion for distinguishing the brain (including ganglia of chelicerae) from the subesophageal ganglion. We also determined the surface of the neuropil of the CNS (the brain and subesophageal ganglion), of the brain and of the arcuate body. We multiplied the surfaces (in mm^2^) by their thickness to obtain the total volume of all elements (in mm^3^). Subsequently, the measurements were used to determine the following: 1/proportion of the brain volume in the CNS volume, 2/proportion of the brain neuropil volume in the brain volume, 3/proportion of the AB volume in the CNS neuropil volume, 4/proportion of the AB volume in the brain volume and 5/proportion of the AB volume in the brain neuropil volume.

To find allometric relationships between the volumes of the particular parts of the CNS, the reduced major axis regression (RMA) was applied to the log-transformed data (with all the developmental stages included). The slope of such a regression line is equivalent to the exponent (*b*) of the allometric equation *y* = *ax*^*b*^. The exponents of the allometric equations (allometric growth coefficients *b*) were compared with the theoretical value of 1, corresponding to the isometric growth. The significance tests of the departure of *b* coefficients from 1 were based on the test statistic according to McArdle (1987):

$$ T = \frac{{\left| {\log b - \log \beta^{\prime } } \right|}}{{\left( {\left( {1 - r^{2} } \right)\left( {n - 2} \right)} \right)^{1/2} }}\;\;{\text{with}}\;df = 2 + \left( {\left( {n - 2} \right)\Big/\left( {1 + 0.5r^{2} } \right)} \right) $$

where *b*—the allometric growth coefficient (RMA regression slope), *β*′—the isometric value of the coefficient (here: 1), *r*—the Pearson correlation coefficient and *n*—the number of data points.

Exponents different from 1 indicate a positive (*b* > 1) or negative (*b* < 1) allometric growth. We checked the relationships between the volume of the arcuate body and the volume of: (1) the total CNS (2) the CNS neuropil, (3) the brain and (4) the brain neuropil.

## Results

In *E. atrica* cultured in laboratory inter-molt periods varied in length. Larvae molted 5–6 days from hatching, whereas nymph stages lasted from 12 (nymph I) to 55 days (nymph IX) on average. Nymphs I and next stages were active, built webs and fed on provided food. Each molt was accompanied by an increase in the volume of the CNS and its components (Table 1).

**Table 1:** Average volume (± standard deviation) of the central nervous system (CNS) and its components, and average proportion of the volume of: the brain in the CNS volume, the brain neuropil in the volume of the brain, the AB in the CNS neuropil volume, the AB in the brain volume and the AB in the brain neuropil volume (*N* = 10)

	*L*	NI	NII	NIII	NIV	NV	NVI	NVII	NVIII	NIX	NX
Average volume of the CNS	460 ± 40	530 ± 60	1010 ± 160	1230 ± 90	1270 ± 150	1900 ± 270	2460 ± 260	2890 ± 410	4070 ± 300	6130 ± 290	8790 ± 600
Average volume of the neuropil of the CNS	150 ± 20	170 ± 20	420 ± 30	550 ± 50	620 ± 60	1120 ± 200	1520 ± 170	1850 ± 260	2660 ± 240	4040 ± 230	5830 ± 450
Average volume of the brain	210 ± 20	220 ± 40	350 ± 70	370 ± 70	360 ± 70	460 ± 90	480 ± 90	510 ± 110	700 ± 60	930 ± 170	1330 ± 200
Average volume of the neuropil of the brain	70 ± 10	60 ± 10	130 ± 10	150 ± 30	170 ± 20	260 ± 40	280 ± 60	310 ± 60	390 ± 50	540 ± 120	810 ± 130
Average volume of the AB	4 ± 1	4 ± 1	9 ± 1	11 ± 1	10 ± 2	14 ± 1	18 ± 1	22 ± 6	25 ± 4	24 ± 2	38 ± 2
Proportion of the volume of the brain in the volume of the CNS	46.33 ± 4.14	40.49 ± 4.51	35.04 ± 5.86	29.51 ± 5.03	28.67 ± 3.34	24.21 ± 2.74	19.71 ± 3.93	17.52 ± 2.64	17.21 ± 1.69	15.17 ± 2.76	15.26 ± 2.60
Proportion of the volume of the neuropil of the brain in the volume of the brain	30.70 ± 5.15	25.44 ± 4.33	38.53 ± 6.87	41.85 ± 6.68	47.68 ± 6.32	57.04 ± 4.34	59.01 ± 1.87	61.49 ± 5.23	56.40 ± 3.76	58.27 ± 5.93	61.12 ± 4.21
Proportion of the volume of the AB in the volume of the neuropil of the CNS	2,72 ± 0.4	2.38 ± 0.55	2.05 ± 0.25	1.97 ± 0.34	1.62 ± 0.32	1.28 ± 0.15	1.22 ± 0.22	1.19 ± 0.30	0.95 ± 0.15	0.60 ± 0.03	0.63 ± 0.08
Proportion of the volume of the AB in the volume of the brain	1.91 ± 0.37	1.84 ± 0.42	2.47 ± 0.51	3.03 ± 0.56	2.87 ± 0.86	3.12 ± 0.48	3.76 ± 1.16	4.36 ± 0.71	3.57 ± 0.36	2.63 ± 0.22	2.93 ± 0.21
Proportion of the volume of the AB in the neuropil of the brain	6.47 ± 1.83	7.28 ± 1.68	6.56 ± 0.66	7.29 ± 1.03	5.99 ± 1.55	5.48 ± 0.73	6.64 ± 1.37	7.19 ± 1.54	6.35 ± 0.69	4.69 ± 1.23	5.03 ± 0.75

The volumetric data are given in mm^3^ × 10^−4^ and proportions in %; (*L* = larva, NI − NX = nymph I − nymph X)

From the larval to nymph X stage, the volume of the CNS increased approximately 19 times and that of its neuropil approximately 39 times. The volume of the brain increased approximately six times: in larvae, it constituted nearly 50% of the volume of the CNS, whereas in nymphs X, only about 15%. The proportion of the brain neuropil in the brain volume increased with each molt, constituting over 60% in nymphs X.

**Fig. 3:**
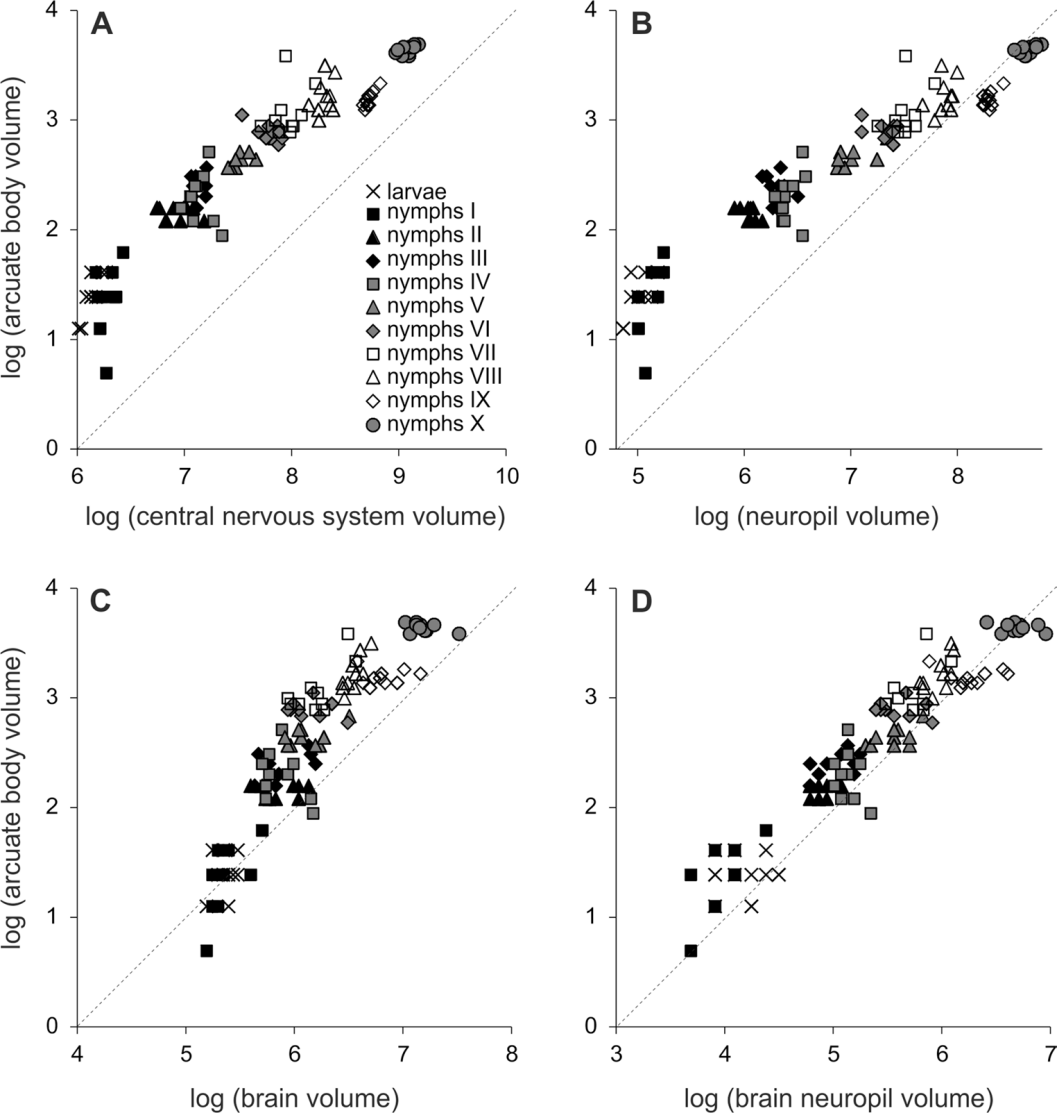
Relationship between the volume of the arcuate body and the volume of: **a** the total central nervous system, **b** total neuropil, **c** brain and **d** brain neuropil during the postembryonic development of *Eratigena atrica*. Ten individuals of each developmental stage were measured

The AB in *E. atrica* was clearly visible in all developmental stages (Fig. 1) and grew within the entire postembryonic development. Interestingly though, it had the same volume in larvae and nymphs I (Table 1, Fig. 3). Its increase was recorded only in nymphs II and later stages. In total, the arcuate body increased its volume more than nine times. Nevertheless, this area developed more slowly than the entire central nervous system and its neuropil, as indicated by the negative results of allometric tests (Table 2, Fig. 3a, b). Because of the intense growth of the CNS neuropil, the proportion of this body in the CNS neuropil volume decreased more than four times. However, positive results of allometric tests indicate that the AB developed faster than the brain (Table 2, Fig. 3c). It constituted 1.84% of the brain in nymphs I and 4.36% in nymphs VII (Table 1). On the other hand, the volume of the AB was negatively allometrically related to the volume of the brain neuropil (Table 2, Fig. 3d), indicating that this body developed much more slowly than the other neuropil areas in the brain. The proportion of the AB in the brain neuropil volume varied, ranging from 4.69% in nymphs IX to 7.29% in nymphs III (Table 1).

**Table 2:** Allometric relationships (reduced major axis regression, RMA) between the volume of the arcuate body (AB) and the volumes of various structures (*X*) of the central nervous system (CNS) of *Eratigena atrica*

CNS part (*X*)	Pearson correlation coefficient			Parameters of the RMA: log (AB) = *B* log (*X*) + *A*		Isometry test: *H*0: *B* = 1	
	*r*	*t*_108_	*P*	*B* ± SE	*A* ± SE	*t*_110_	*P*
Total CNS	0.954	33.06	< 0.001	0.798 ± 0.023	− 3.456 ± 0.175	7.83	< 0.001
Total neuropil	0.967	39.38	< 0.001	0.623 ± 0.015	− 1.718 ± 0.107	19.28	< 0.001
Brain	0.906	22.23	< 0.001	1.294 ± 0.053	− 5.366 ± 0.324	6.33	< 0.001
Brain neuropil	0.957	34.18	< 0.001	0.890 ± 0.025	− 2.214 ± 0.135	4.15	0.001

The isometry test shows whether the slope of the RMA line (*b*) significantly departs from 1, i.e. whether the AB grows slower (*B* < 1) or faster (*B* > 1) than the given part of the CNS

## Discussion

Our study confirms the hypothesis of allometric growth of the arcuate body in subsequent stages of *Eratigena atrica*. The results indicate that the arcuate body increased ten times within the postembryonic development. Interestingly, however, in two initial developmental stages, the volume of body did not increase. It was only in nymphs II that it grew rapidly, doubling in size and marking the biggest increase in relation to the previous stage. It can thus be concluded that there is a relationship between spiders leaving the cocoon and starting an active lifestyle and the enlargement of the AB, which suggests its coordinating and associative functions. This is also confirmed by the connection of the AB to the subesophageal ganglion through motor fibres (e.g. a large protocerebro-dorsal tract) (Babu and Barth 1984; Foelix 1996; Barth 2002; Park et al. 2013).

Weltzien and Barth (1991) described the development of the AB in the postembryogenesis of two spider species with different behaviour, i.e. orb-weaver *Nephila clavipes* and wandering *Cupiennius salei*. Their results show that even in the earliest stages (remaining in the cocoon), this body could be easily distinguished. Our results coincide with the above observation: we found the AB in both larvae and nymphs I of *E. atrica*. This leads to the conclusion that it is present in spiders regardless of their developmental stage and lifestyle. This contradicts the observation of Babu (1975) that the AB was not yet differentiated in the earliest stages of *A. aurantia* and was fully formed only when spiders started weaving webs. According to Babu (1975), this neuropil mass is correlated with this particular activity. Even if this is true for this species, it does not seem to be a general rule for all spider taxa.

**Fig. 4:**
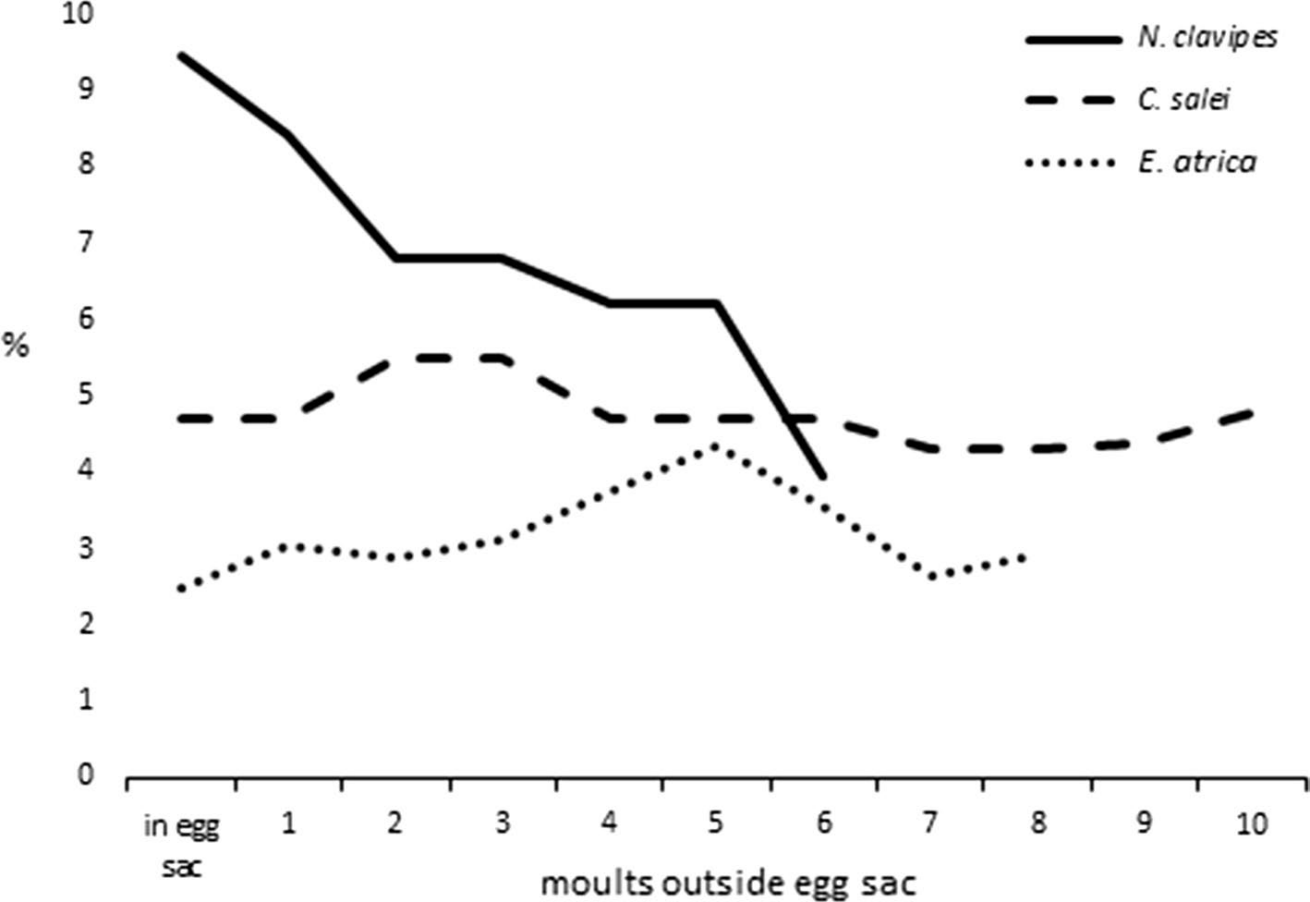
Models of development of the arcuate body in postembryogenesis (expressed as changes in the proportion of the AB volume in total brain volume) in three species of spiders differing in behaviour: *N. clavipes, C. salei* (based on Weltzien and Barth 1991) and *E. atrica* (our study)

In our study, the proportion of the AB in the CNS of *E. atrica* and its neuropil volume decreased gradually, whereas it developed faster than the brain itself, confirming its vital role in the spider biology. Comparative analysis indicates that the AB in *E. atrica* develops differently than in other studied species (Weltzien and Barth 1991). In the orb-weaver *N. clavipes*, the earliest stages had a relatively large AB compared to the brain (over 9% share). However, this proportion decreased in subsequent stages, before they started to build webs, to be the lowest in adult specimens (3.97%). In the wandering *C. salei,* the proportion of the AB in the brain volume was similar through the entire postembryogenesis: 4.70% in the earliest stages and 4.80% in adults (Fig. 4). Our results show that the model of growth of the AB in *E. atrica* is different than in the orb-weaver spider and wandering spider. Eventually, the final proportion of the AB in the brain volume in nymphs X of *E. atrica* was similar to that of the adult stage of the jumping spider *Phidippus regius* (3.1%) (Weltzien and Barth 1991). However, the data on the earlier stages of this species are not known.

In general, it seems justified to state that in spiders, the AB occupies a relatively small part of the brain, in adults never exceeding several per cent of its volume. Therefore, the results presented by Satija et al. (1970a) suggesting 35% proportion of the arcuate body in the brain volume of *Oecobius putus* and *Filistata poonaensis* seem questionable. Research on other spider taxa is needed to clarify this issue.

The development of the body and its organs can be associated with their functional role in animal behaviour. This also applies to the central nervous system and its structures in spiders. Their AB was analysed in the context of web building (mainly orb webs) (Hanström 1926, 1928 quoted after Weltzien and Barth 1991; Babu 1975). If the AB was indeed the centre of web-weaving behaviour, web-weaving spiders, such as *N. clavipes*, should boast the largest AB (their web construction requires precision, motor skills and coordination). On the other hand, this structure would be smaller in wandering and jumping spiders. The results, however, contradict this hypothesis. Nevertheless, the influence of the AB on silk spinning cannot be completely denied, especially since silk can also be used for nest and cocoon construction. *Eratigena atrica* occupies an intermediate position between the behaviour types shown by the above-mentioned species: it builds webs, though not as complex as typical orb weavers, as well as wanders actively in search of food. Therefore, on the basis of our results and the above-mentioned literature data, it is not possible to associate the dimensions of the AB with one particular activity. Instead, it is likely that it is involved in several behaviour types, perhaps to various extents in various species. Perhaps this is why its proportion in the total brain volume increases with age in *E. atrica*, the species exhibiting both high movement and web-weaving activity. In jumping spiders, strongly depending on their vision, the AB may play yet another role, being an optical centre (Babu and Barth 1984; Babu 1985; Foelix 1996). This suggests a visual function of this body.

**Fig. 5:**
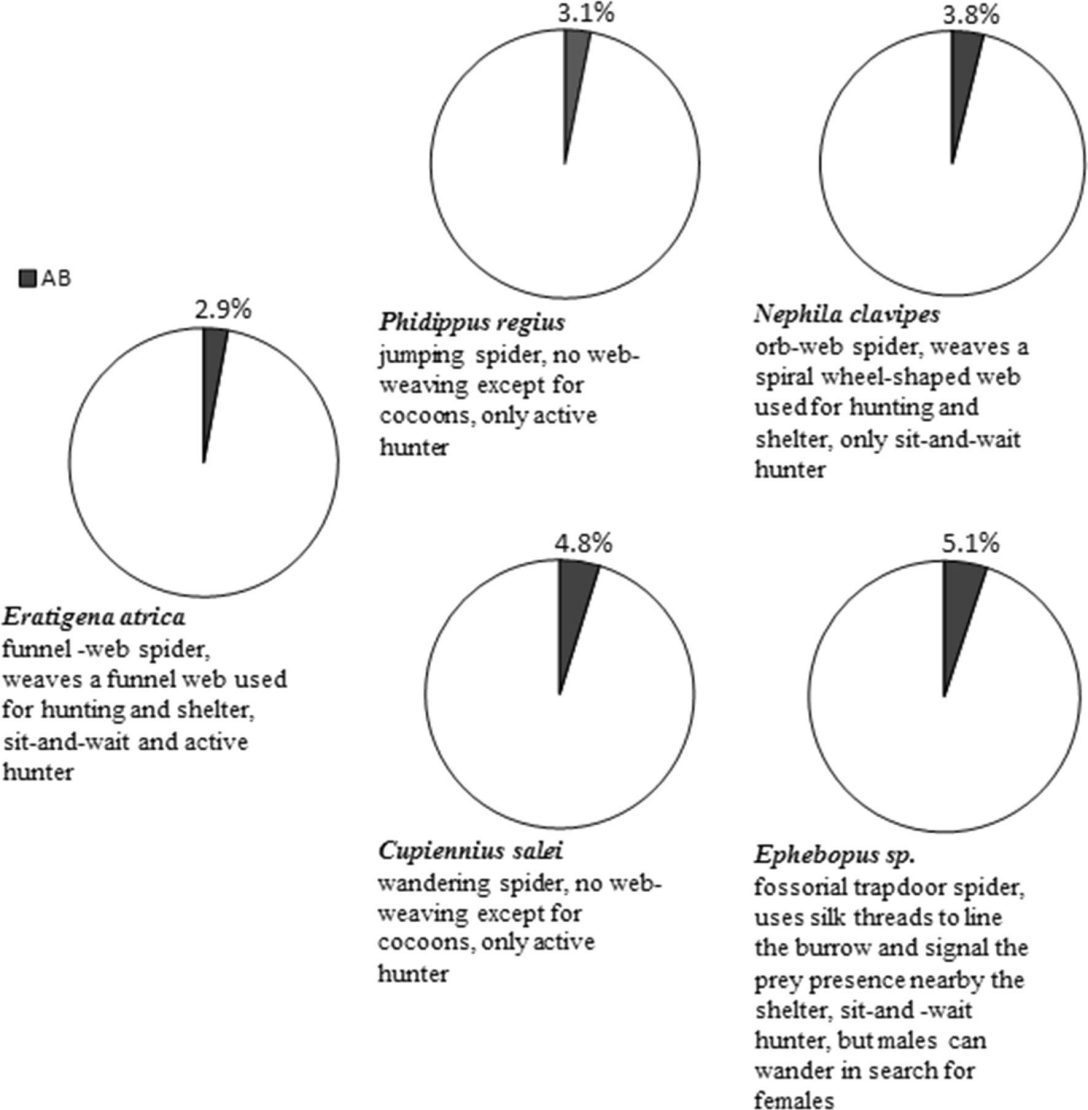
Volume of the arcuate body of the spider brain given as the percentage of the total brain volume (100%) in five spider species differing in behaviour: *N. clavipes, C. salei*, *P. regius*, *Ephebopus* sp. (based on Weltzien and Barth 1991) and *E. atrica* (our study). *AB* arcuate body

In conclusion, our allometric study of the AB in *Eratigena atrica* provides new information on the development of this neuropil mass in the postembryogenesis of spiders. Taking into account previous and present research, we conclude that there is no universal model of how this body develops in these arachnids. However, in adult spiders, regardless of their biology and behaviour, the proportion of the AB in the brain is similar and constitutes several per cent of its volume (Fig. 5).
